# Guidelines (1988) for preparation of laboratory procedure manuals for clinical chemistry

**DOI:** 10.1155/S1463924689000076

**Published:** 1989

**Authors:** C. G. Fraser, T. D. Geary, H. G. J. Worth

**Affiliations:** 1 Ninewells Hospital and Medical School Dundee UK; 2 Institute of Medical and Veterinary Science Adelaide Australia; 3 King's Mill Hospital Nottinghamshire Sutton-in-Ashfield NG17 4JL UK


Journal of Automatic Chemistry Vol. 11, No. 1 (January–February 1989), pp. 32–35

### Guidelines (1988) for preparation of laboratory procedure manuals for clinical chemistry

### International Federation of Clinical Chemistry (IFCC)† Education Committee, Expert Panel on Instrumentation and International Union of Pure and Applied Chemistry,† Commissions on Automation and Clinical Techniques and Teaching. IFCC document stage 2, draft 3

**Prepared for publication by:**

**C. G. Fraser**
*Ninewells Hospital and Medical School, Dundee, UK*

**T. D. Geary**
*Institute of Medical and Veterinary Science, Adelaide, Australia*

**H. G. J. Worth**
*King's Mill Hospital, Sutton-in-Ashfield, Nottinghamshire NG17 4JL, UK*

*Comments on the Guidelines and reprint requests should be sent to Dr H. G. J. Worth.*
*Comments from the viewpoint of languages other than English are encouraged.*

*Education Committee members: O. Zinder (IL), Chairman; H. G. J. Worth (GB), Secretary; N. de Cediel (CO); A. Deom (CH); D. G. Fraser (GB); L. Josefsson (DK), representative of the International Union of Biochemistry.*

*Expert Panel Members: R. Haeckel (DE), Chairman; T. D. Geary (AU), Secretary; P. Bonini (IT); C. A. Burtis (US).*

*Membership of the Commission on Automation and Clinical Chemical Techniques during the period (1985–7) in which these Guidelines were prepared: R. Haeckel (DE), Chairman; T. D. Geary (AU), Secretary. Titular members: P. Bonini (IT); C. A. Burtis (US). Associate members: J. Bierens de Haan (CH); M. Hjelm (GB). National representatives: E. A. Etchegarey (AR); R. D. Baillie (CA); C. Naudin (FR); V. Haviaras (GR); P. P. Juhasz (HU); F. Salvatore (IT); K. Okuda (JP); S. H. Strand (NO); S. Angielski (PO); A. Hornfelt (SE); N. Gochman (US).*

*Membership of the Teaching Commission during the period (1985–7) in which these Guidelines were prepared: O. Zinder (IL), Chairman; H. G. J. Worth (GB), Secretary. Titular member: C. G. Fraser (GB). Associate members: M. A. Drosdowsky (FR); P. Garcia Webb (AU); N. Montalbetti (IT); C. J. Porter (CA); B. Straus (YU); V. N. Titov (SU); R. Vihko (FI); W. H. C. Walker (CA). National representative: M. M. Abel Kader (EG); J. Agneray (FR); K. Bergström (SE); B. Christophersen (NO); A. F. Delbruk (DE); H. A. Fritsche Jr (US); A. Gornall (CA); A. G. Hadjivassiliou (GR); T. Kanno (JP); M. Nemeth Csoka (HU); P. Strom (IT).*

† The exclusive © for all languages and countries is vested in the International Federation of Clinical Chemistry.

### 1. Introduction

The satisfactory training of laboratory staff [1 and 2] and medical students [3] can be facilitated by the use of detailed laboratory procedure manuals. Laboratory procedure manuals are also required because staff performing analytical work may make major or minor changes to a method in the hope that the results will be more reliable or can be obtained with less effort, or in a shorter time. Unfortunately, such modifications may lead to a deterioration of the quality of analytical results produced. It is therefore good practice that detailed procedures for all methods are documented and followed exactly. Also, some countries have legislation which requires that comprehensive instructions for analytical methods are available to laboratory staff at their work stations.

There is little information available on the preparation and use of laboratory procedure manuals in clinical chemistry. The National Committee for Clinical Laboratory Standards of the United States of America, through the Sub-Committee on Procedure Manuals of the Area Committee on Clinical Laboratory Administration and Labelling, has prepared an excellent document [4] on this subject. However, this document addresses the US laboratory community in general and is not related specifically to clinical chemistry. Also, although the National Committee for Clinical Laboratory Standards has a large membership, particularly in the United States of America, this document is not widely available throughout the world.

The IUPAC Commission on Teaching of Clinical Chemistry/IFCC Education Committee and the IUPAC Commission on Automation and Clinical Chemical Techniques/IFCC Expert Panel on Automated Systems have documented guidelines for the preparation of laboratory procedure manuals for clinical chemistry which are applicable to all laboratories, irrespective of their size or geographical location. These guidelines are detailed here and, to some extent, are based upon the recommendations of the National Committee for Clinical Laboratory Standards [4].

0142–0453/89 $3.00 © 1989 Taylor & Francis Ltd.

### 2. Scope of a laboratory procedure manual

In order to achieve their aims of facilitating education and training and of ensuring the consistent quality of analytical results, laboratory procedure manuals should cover, in detail, all aspects of every analytical test performed in the laboratory. Thus, laboratory procedure manuals should contain full information on the following:

(i) Clinical indications for the test.

(ii) Specimen requirements, collection procedures, information on appropriate conditions of storage and relevant disposal procedures.

(iii) Principles of the analytical method.

(iv) Preparation of reagents, standards or calibrators, quality control materials and details of how the analytical procedure is performed.

(v) Full details of instrument maintenance protocols if specific instrumentation is used.

(vi) Reporting of results and clinical interpretation.

(vii) Other pertinent data.

### 3. Detailed contents

The full information required in the manual is as follows:

*3.1. Clinical details*

The manual should contain brief information on the uses of the test results. The main categories are:

(i) Diagnosis, including confirmation of clinical signs or symptoms (which should be briefly summarized), and aiding in differential diagnosis.

(ii) Monitoring of treatment or therapy.

(iii) Assessment of prognosis.

(iv) Detection of disease in the apparently healthy (screening).

(v) Other aspects of clinical care.

When possible, mention of the clinical sensitivity, specificity and predictive value of the test should be made, if possible for each of the modes in which the test is used.

*3.2. Specimen requirements and collection procedures*

The manual should detail the following points:

*3.2.1.* Any special requirements for patient preparation including (i) time of day specimen should be collected; (ii) period of collection if a timed urine or faeces specimen is required; (iii) requirements for fasting or avoidance of certain foodstuffs, drinks or drugs; (iv) posture to be adopted; and (v) time in relation to onset of acute disease process.

*3.2.2.* The specimen types that are suitable and unsuitable; preferred and unacceptable anticoagulants, preservatives and stabilizers; generally required and minimally acceptable specimen volumes.

*3.2.3.* Details of specimen handling and preparation, for example, requirement for refrigerated centrifugation.

*3.2.4.* Analyte stability and conditions of specimen storage, period for which specimens should be retained after analysis and method of disposal.

*3.2.5.* The action to be taken should the specimen provided be unsatisfactory for analysis.

*3.3. Principles of analytical technique*

The chemical, biochemical and instrumental principles should be briefly noted. Reaction sequences and formulae can be advantageously included. It is appropriate here to review (i) possible interference due to lipaemia, icterus and haemolysis and drug effects (*in vivo* and *in vitro*); (ii) foodstuffs or drinks which may cause problems; (iii) interference or cross-reaction by other compounds or metabolites which occur naturally in both health and disease states; and (iv) problems which arise from use of inappropriate collection, preservation and stabilization of specimens.

*3.4. Performance of the analytical technique*

The following should be documented:

*3.4.1.* The full analytical procedure should be detailed in a sequential manner and all steps made totally unambiguous with regard to each of the points detailed below. In addition, suitable ancillary instrumentation (for example, water-baths, centrifuges, spectrometers and beta- and gamma-radioisotope counters) must be fully specified and all relevant details of their operation and maintenance should be given (see Section 3.5), if specific to the test under discussion.

*3.4.2.* Clear instructions should be given as to the corrective action required should the criteria for acceptability of performance not be met.

*3.4.3.* Relevant safety precautions to be taken should be detailed and highlighted at the appropriate points.

*3.4.4.* All glassware, disposables and pipetting devices used should be detailed, and acceptable and unacceptable alternatives given. Cleaning requirements should be documented.

*3.4.5.* Names, formulae, acceptable and unacceptable grades, suppliers and catalogue numbers of all reagents should be given.

*3.4.6.* Reagent preparation, glassware type and explicit directions on preparation should be given. Quality control of reagents, such as measurements of pH and absorbance, with criteria of acceptability, and methods of storage of reagents with details of appropriate and inappropriate containers and temperatures should all be documented. Details of shelf life are also required.

*3.4.7.* The points outlined for reagents (*3.4.5.* and *3.4.6.* above) should also be detailed for standards or calibrators and quality control materials used.

*3.4.8.* The procedure for the use of standard(s) or calibrator(s) should be detailed, including an example of the graph obtained if this is the usual mode of standardisation or calibration. Details of the criteria of acceptability and the limitations of any algorithms or calculations used are also required.

*3.4.9.* The use of quality control materials, including frequency of analysis, position(s) in analytical batches and setting of criteria for acceptance or rejection of runs, should be detailed.

*3.4.10.* If calculations are required to convert data into results, full instructions, complete with examples and methods of dealing with dilutions and out of range readings, should be given.

### *3.5. Instrument operating and maintenance protocols*

If the instrumentation is specifically used for the test, information should be included on the following points:

*3.5.1.* Date of purchase, warranty period, and from whom purchased.

*3.5.2.* Operating instructions.

*3.5.3.* Instrument performance data and characteristics.

*3.5.4.* Service checks and preventative maintenance.

*3.5.5.* Procedure for recording the date on which and by whom calibrations, repairs and maintenance (including cleaning and decontamination) have been carried out.

*3.5.6.* Cleaning and decontamination instructions.

*3.5.7.* Effect of electrical power fluctuations.

*3.5.8.* Temperature and humidity operating ranges.

Where an instrument is used in several different analyses, there should be a separate laboratory procedure document for the above points; this can then be referred to in the appropriate method descriptions.

### *3.6. Reporting of results and clinical interpretation*

*3.6.1.* Acceptable reporting styles should be detailed, including the logical rounding of results. The units used should ideally conform to the SI system [5] but generally accepted local, regional or national recommendations or practice should be followed.

*3.6.2.* The relevant guidelines prepared by the IFCC Education Committee on the reporting of laboratory results [6] should be applied. Pertinent points are that details on the use of terminology, abbreviations, units and symbols should be given in the laboratory procedure manual to ensure consistency. Information should also be given on the requirements for confidentiality, scrutiny of completed reports, mechanisms of reporting results (both prior to verification and in emergency situations) and aceptability or otherwise of turn-around times. Details of procedures for rapid reporting of extreme values of real clinical urgency should be documented.

*3.6.3.* Reference values should be detailed and the guidelines of the IFCC Expert Panel on Theory of Reference Values (EPTRV) [7] followed, i.e. reference values should only be reported if it can be ensured that they are relevant to the observed value found. Hence different reference values should be reported for groups which differ with regard to factors such as age, sex, posture, activity etc. If this cannot be achieved, the approach recommended by the EPTRV is to confine reference value data to documents providing information to laboratory and clinical staff, namely, the laboratory procedure manual and laboratory handbook, respectively.

*3.6.4.* A reasonably detailed indication of the clinical meaning of results should be given.

### *3.7. Other pertinent points*

The laboratory procedure manual should give, following each procedure, a small number of the most relevant literature references covering both methodological and clinical aspects of the test. Further information of a technical nature, and expansion of the material detailed above which is considered of educational value, should be described in this section.

### 4. Administrative details

### *4.1. Styles*

The exact style to be used for the laboratory procedure manual is a matter for the individual laboratory. Looseleaf folders have advantages in that replacement of pages or sections is easy and different areas of the laboratory can each be given information relevant to their needs, although one or more full master copies should be maintained. The use of plastic covers or plastic folder inserts facilitates the use of specific procedures at the laboratory bench. The title pages of each procedure, numbering of pages and semantic style should be as consistent as possible. In laboratories with appropriate facilities, word processing systems can make the creation, updating and printing of the procedure manual an easier and less time-consuming task; data security is important in this situation to ensure that unauthorized changes were not made.

### *4.2. Review and updating*

Each procedure should be reviewed at regular intervals, perhaps annually, although minor updating must take place as a continuous process. Review and updating is required whenever a significant change in methodology or instrumentation is introduced. Each laboratory must decide which staff members are allowed to review and update the manuals. Any minor alteration to the laboratory procedure should be made as an addition to the text; the original procedure should not be removed until a major review is performed. An archive of the outdated procedures should be retained for reference and the rationale for the modifications made filed. Each procedure should contain the date of establishment of the procedure, the date of the last review and updating and the names of the staff members involved in these.

### *4.3. Reagent kit set inserts and other literature*

Package inserts from reagent kit sets and recipes from textbooks are not suitable for use in laboratory procedure manuals because much of the information, for example, on reference values, performance characteristics and clinical utility, is of a general nature and may not be relevant to the individual laboratory. Such materials, if kept up to date, may be inserted in the laboratory procedure manual as appendices to the text.

### 5. Conclusions

Laboratory procedure manuals have a crucial role to play in the education and training of staff and students and in the maintenance of production of high quality results. The guidelines detailed here should help all laboratories to prepare these manuals.

### 6. References

1. Porter, C. J. and Curnow, D. H., *Pure and Applied Chemistry,* **55** (1983), 557; *Journal of Clinical Chemistry and Clinical Biochemistry,* **21** (1983) 185; *Clinica Chimica Acta,* **131** (1983) 351F.
2. Worth, H. G. J., Curnow, D. H., Fraser, C. G., Porter, C. J., Schwartz, M. K. and Zinder, O. *Pure and Applied Chemistry* **56** (1984), 1505; *Journal of Clinical Chemistry and Clinical Biochemistry,* **22** (1984), 497; *Clinical Chemistry Newsletter,* **2–4** (1984), 98.
3. Fraser, C. G., Zinder, O., de Cediel, N., Porter, C. J., Schwartz, M. K. and Worth, H. G. J., *Journal of Clinical Chemistry and Clinical Biochemistry,* **23** (1985), 697; *Clinical Chemistry Newsletter,* **2–4** (1985), 120.
4. National Committee for Clinical Laboratory Standards, *Approved Guidelines for Clinical Laboratory Procedure Manuals,* Volume 4, No. 2 (National Committee for Clinical Laboratory Standards, Villanova, PA., 1984).
5. Dybkaer, R., *Pure and Applied Chemistry,* **37** (1974), 549.
6. Fraser, C. G., de Cediel, N., Porter, C. J., Schwartz, M. K., Worth, H. G. J. and Zinder, O., *Journal of Clinical Chemistry and Clinical Biochemistry,* **23** (1985), 891.
7. Dybkaer, R., *Journal of Clinical Chemistry and Clinical Biochemistry,* **20** (1982), 841; *Clinica Chimica Acta,* **127** (1982), 331F; *Chimica Newsletter,* **3** (1982), 115; *Clinical Biochemistry,* **15** (1982), 237.

### NOTES FOR AUTHORS

*Journal of Automatic Chemistry* covers all aspects of automation and mechanization in analytical, clinical and industrial environments. The *Journal* publishes original research papers; short communications on innovations, techniques and instrumentation, or current research in progress; reports on recent commercial developments; and meeting reports, book reviews and information on forthcoming events. All research papers are refereed.

### Manuscripts

Two copies of articles should be submitted. All articles should be typed in double spacing with ample margins, on one side of the paper only. The following items should be sent: (1) a title-page including a brief and informative title, avoiding the word 'new' and its synonyms; a full list of authors with their affiliations and full addresses; (2) an abstract of about 250 words; (3) the main text; (4) appendices (if any); (5) references; (6) tables, each table on a separate sheet and accompanied by a caption; (7) illustrations (diagrams, drawings and photographs) numbered in a single sequence from 1 upwards and with the author's name on the back of every illustration; captions to illustrations should be typed on a separate sheet. Papers are accepted for publication on condition that they have been submitted only to this *Journal.*

### References

References should be indicated in the text by numbers following the author's name, i.e. Skeggs [6]. In the reference section they should be arranged thus:

to a journal

Manks, D. P., *Journal of Automatic Chemistry,* **3** (1981), 119.

to a book

Malmstadt, H. V., in *Topics in Automatic Chemistry,* Ed. Stockwell, P. B. and Foreman, J. K. (Horwood, Chichester, 1978), p. 68.

### Illustrations

Original copies of diagrams and drawings should be supplied, and should be drawn to be suitable for reduction to the page or column width of the *Journal,* i.e. to 85 mm or 179 mm, with special attention to lettering size. Photographs may be sent as glossy prints or as negatives.

### Proofs and offprints

The principal or corresponding author will be sent proofs for checking and will receive 50 offprints free of charge. Printers errors only may be corrected; any other amendments will be charged to the author. Additional offprints may be ordered on a form which accompanies the proofs.

*Manuscripts should be sent to Dr P. B. Stockwell, P.S. Analytical Ltd, Arthur House, Unit B4, Chaucer Business Park, Watery Lane, Kemsing, Sevenoaks, Kent TN15 6QY, UK.*

